# Process development for the elucidation of mycotoxin formation in *Alternaria alternata*

**DOI:** 10.1186/2191-0855-1-27

**Published:** 2011-10-04

**Authors:** Katrin Brzonkalik, Tanja Herrling, Christoph Syldatk, Anke Neumann

**Affiliations:** 1 Institute of Process Engineering in Life Sciences, Section II: Technical Biology, Karlsruhe Institute of Technology, Engler-Bunte-Ring 1, D-76131 Karlsruhe, Germany

**Keywords:** *Alternaria alternata*, Mycotoxin, Batch process, Aeration rate

## Abstract

The black mould *Alternaria alternata *produces a wide diversity of mycotoxins which are of particular health concern. Since no maximum allowable limits are set for *Alternaria *toxins in food and feed, prevention of *Alternaria *infestations and mycotoxin spoilage is the only way to avoid health risks. Thus, the understanding of mycotoxin biosynthesis is essential. For that purpose, a reliable batch process in a 2 L bioreactor was established which enables the study of several parameters influencing the production of the mycotoxins alternariol (AOH), alternariol monomethylether (AME) and tenuazonic acid (TA) by *A. alternata *DSM 12633. Modified Czapek-Dox medium was used with glucose as carbon source and ammonium and nitrate as nitrogen sources. Consumption of carbon and nitrogen sources as well as formation of the three mycotoxins were monitored; the average data of five independent fermentations was plotted and fitted using a logistic equation with four parameters. Maximum mycotoxin concentrations of 3.49 ± 0.12 mg/L AOH, 1.62 ± 0.14 mg/L AME and 38.28 ± 0.1 mg/L TA were obtained.

In this system the effect of different aeration rates (0.53 vvm-0.013 vvm) was tested which exerted a great influence on mycotoxin production. The use of the semi-synthetic Czapek-Dox medium allowed the exchange of carbon and nitrogen sources for acetate and aspartic acid. The use of acetate instead of glucose resulted in the sole production of alternariol whereas the exchange of ammonium and nitrate for aspartate enhanced the production of both AOH and AME while TA production was not affected.

## Introduction

Mycotoxins are secondary metabolites of low molecular weight produced by filamentous fungi. Since the discovery of the first mycotoxins, the aflatoxins, in 1960 which caused the death of 10,000 turkeys many new mycotoxins have been identified in the last 50 years. Today 300 to 400 compounds are designated as mycotoxins (Bennett and Klich 2003). As other secondary metabolites mycotoxins are formed subsequently to the growth phase and are not necessary for growth or development (Fox and Howlett 2008). Mycotoxin formation is subjected to a complex regulation, but it is often induced by nutrient limitation (Demain 1986). Mycotoxins are released by the fungus in the surrounding substrate and contamination of agricultural products is therefore possible. They are connected to certain health disorders and elicit acute toxic, mutagenic, teratogenic, carcinogenic and sometimes estrogenic properties (Bhatnagar et al. 2002). Based on estimations of the Food and Agriculture Organization (FAO) of the United Nations approximately 25% of the world's food crops are affected by mycotoxin producing fungi and global losses of foodstuffs due to mycotoxins are in the range of 1000 million tons per year http://www.fao.org/ag/agn/agns/chemicals_mycotoxins_en.asp.

*Alternaria *species are wide spread black moulds which belong to the division of Deuteromycota (Bottalico and Logrieco 1998) and are common saprophytes found on decaying organic material world-wide. The genus *Alternaria *includes also opportunistic plant-pathogens affecting many cultivated plants in the fields and stored fruits and vegetables during post-harvest (Guo et al. 2004). *Alternaria *species are capable to produce a wide diversity of secondary metabolites belonging to different chemical groups including dibenzopyrones, tetramic acids, lactones, quinones and cyclic peptides. More than 120 secondary metabolites of *Alternaria *species are known; a quarter of that is designated as mycotoxins (Panigrahi 1997). Five major *Alternaria *toxins can be found as natural contaminants in foodstuffs: the benzopyrene derivatives alternariol (AOH), alternariol monomethylether (AME), altenuene (ALT), the tetramic acid tenuazonic acid (TA) and the perylene derivative altertoxin I (ATX I) (Barkai-Golan 2008). These toxins were detected in apples (Stinson et al. 1981), tomatoes (Stinson et al. 1981), wheat ([Bibr B2][Bibr B23][Bibr B34]), olives (Visconti et al. 1986), sunflower seeds (Pozzi 2005), fruit juices (Lau et al. 2003) and tomato products ([Bibr B31][Bibr B51]). Therefore, *Alternaria *toxins can be considered as toxic contaminant of our everyday food (Barkai-Golan 2008).

Although the acute toxicity of *Alternaria *toxins is low (LD_50 _of *Alternaria *extracts: 300 mg/kg body weight in mice, LD_50 _of AOH: > 400 mg/kg body weight of mice (Pero et al. 1973)), they are connected to certain health disorders. *Alternaria *extracts have been described as mutagenic and tumorigenic ([Bibr B25][Bibr B42]). Of particular health concern is the incidence of esophageal cancer in Linxin, China. The etiology of this cancer was connected with the contamination of cereal grains with *A*. *alternata *([Bibr B12][Bibr B24]). As stated by (Pero et al. 1973), the toxicity of complex extracts is much higher than of the single tested mycotoxins which suggest synergism between single components. Although numerous toxicological studies were conducted to clarify the effects of *Alternaria *toxins a risk assessment is not possible. According to the German Federal Institute of Risk Assessment (Bundesinstitut für Risikobewertung, BfR 2003) only little toxicological data are available just for seven out of the 30 known *Alternaria *mycotoxins which is insufficient for an assessment of the health risk for the consumer.

As long as maximum allowable limits in food for *Alternaria *toxins were not defined prevention of *Alternaria *infestations and mycotoxin spoilage is the best way to avoid health risks. Therefore, knowledge about factors which enhance or inhibit mycotoxin production and its regulation is crucial. Mycotoxin production varies with fungal strain, the substrate and environmental growth conditions. This includes factors like water activity, temperature, pH-value and light. According to (Schmidt-Heydt et al. 2008) mycotoxin production can be regarded as an adaptation to imposed abiotic or other stresses of the mycotoxigenic species. Whereas the influence of water activity, temperature and light was extensively studied for different *A. alternata *strains and media ([Bibr B17][Bibr B26][Bibr B38][Bibr B41][Bibr B45]), the effects of pH and nutritional factors were neglected. Additionally, all these studies use different kinds of media and culture conditions, e.g. solid agar-media, liquid surface culture or drop cultures, which render direct comparisons difficult. The intention of this work is therefore the establishment of a reproducible system which enables the elucidation of all important influences on mycotoxin production in *A. alternata*. For optimal reproducibility and comparability of single experiments a process in a bioreactor was developed allowing direct monitoring and prevention of nutrient or oxygen limitations. With respect to the production of mycotoxins with *Alternaria *spp. submerged fermentation protocols were not developed yet. To the knowledge of the authors only one protocol for submerged fermentation of *Alternaria *spp. for the production of the new antibiotic altersetin (Hellwig et al. 2002) and another solid state fermentation protocol for the production of mycoherbicidal agents with *A. alternata *(Singh et al. 2010) were published. The primary purpose of the presented process is to enhance the knowledge of regulatory mechanisms for *Alternaria *toxin production but additionally it may be helpful for the development of further production processes of other interesting secondary metabolites of *Alternaria *spp.

## Materials and methods

### Strain, media and processing

*A. alternata *DSM 12633 was obtained from DSMZ culture collection ("Deutsche Sammlung von Mikroorganismen und Zellkulturen", Braunschweig, Germany). All cultures of *A. alternata *were routinely grown on PDA (Roth, Germany). Conidia were harvested with 25% glycerol solution from plates that were incubated for seven days at 28°C and filtered through Miracloth (Calbiochem). Conidia were counted in a Thoma counting chamber and diluted with glycerol to a concentration of 1*10^6 ^condia per ml. Aliquots of glycerol stocks were stored at -80°C.

For the fermentation experiments 1.5 L of modified Czapek-Dox medium (modified after Gatenbeck and Hermodsson 1965) at pH 5.5 were used: 10 g/L glucose, 0.06 g/L NH_4_Cl, 0.25 g/L NaNO_3_, 1 g/L KH_2_PO_4_, 0.5 g/L MgSO_4 _* 7 H_2_O, 0.25 g/L NaCl, 0.25 g/L KCl, 0.01 g/L FeSO_4 _* 7 H_2_O, 0.01 g/L ZnSO_4 _* 7 H2O, 1 g/L yeast extract. For the experiments with alternative carbon and nitrogen sources glucose and/or the mixture of ammonium chloride and sodium nitrate were replaced. In a first experiment glucose was exchanged for 22.66 g/L sodium acetate trihydrate and in a second experiment the mixture of ammonium chloride and sodium nitrate was exchanged for 0.54 g/L aspartic acid. In a third experiment both glucose and ammonium chloride/sodium nitrate were exchange for acetate and aspartic acid. In all experiments the same amounts of carbon (4 g/L) and nitrogen (56.8 mg/L) were used. The carbon sources were prepared separately and were added after autoclaving. The process was operated in the small-scale bioreactor (vessel volume 2.0 L) Minifors (Infors, Bottmingen, Switzerland) for the indicated time period at 28°C in the dark. The medium was inoculated directly with a thawed aliquot of 1*10^6 ^conidia, a pre-culture was not used. The bioreactor was equipped with two 6-blade Rushton Turbines; stirring speed was enhanced from 400 rpm to 900 rpm after 48 h. The aeration rate was 0.013 vvm if not indicated otherwise. For pH adjustment 2 M sodium hydroxide and 2 M phosphoric acid were used.

### Analytical methods

#### Data analysis

Nutrient consumption and mycotoxin production were fitted using a logistic equation with four parameters in a scientific data analysis and graphing software (Sigma Plot 9.0, Systat, San Jose, USA). The used equation was:

(1) $$y\left(x\right)=y_{0}+\frac{a}{1+{\left(\frac{x}{x_{0}}\right)}^{b}}$$

The four parameters are the following: y_0 _indicates the minimum concentration of the respective nutrient or mycotoxin; a indicates the maximum nutrient/mycotoxin concentration; x_0 _indicates the process time when half of the nutrient amount is consumed or half of the maximum mycotoxin concentration is produced; b is a shape parameter and difficult to explain biologically (Erkmen and Alben 2002). Ammonium and AME concentration lapse were derived analogously with a logistic equation with three parameters (cf. Eq. 1, but excluding y_0_). Derivation of the fitting was used for the determination of absolute consumption and production rates.

#### Detection of mycotoxins

Alternariol (AOH), alternariol monomethylether (AME) and tenuazonic acid (TA) were analyzed simultaneously by HPLC. The standard HPLC device (Agilent 1100 Series, Agilent, Waldbronn, Germany) was equipped with a 25 cm reversed phase column (Luna 5 μm C18(2), Phenomenex, Aschaffenburg, Germany). Analyzes were performed at 30°C and a flow rate of 0.7 ml/min. Mobile phase solution was methanol/0.1 M NaH_2_PO_4 _(2:1), pH 3.2 (according to Shephard et al. 1991). Mycotoxins were monitored with a UV detector at 280 nm. For quantification a standard curve with mycotoxin standard solutions was prepared. The standards were purchased from Sigma-Aldrich (Munich, Germany) and solved in methanol.

Mycotoxins were extracted twice with equal amounts of ethyl acetate from 5 ml culture broth after acidifying with 5 μl conc. HCl. The supernatants were combined and evaporated to dryness in a vacuum centrifuge. The residue was dissolved in methanol and used for HPLC analyzes. More than 90% of the mycotoxins could be extracted by this method. Retention times were 5.3 ± 0.1 min (TA), 10.2 ± 0.2 min (AOH) and 23.3 ± 0.1 min (AME). Detection limits for this method were 16 ng of injected AOH, 33 ng of injected AME and 12 ng of injected TA.

#### Quantification of nutritional components and biomass

The glucose concentration during the fermentation process was monitored with the photometrical anthrone assay (Pons et al. 1981).

Ammonium and nitrate were determined with the photometrical assays "Ammonium-Test" (Spectroquant^®^, Merck, Darmstadt, Germany) and "Nitrat-Test" (Spectroquant^®^, Merck, Darmstadt, Germany).

For biomass quantification fungal mycelium was transferred from the bioreactor at the end of fermentation to a weighed tube and dried completely at 60°C. The weight was determined on a standard balance.

## Results

### Process parameters of *A. alternata *fermentation in a 2 L bioreactor system

**Figure 1:**
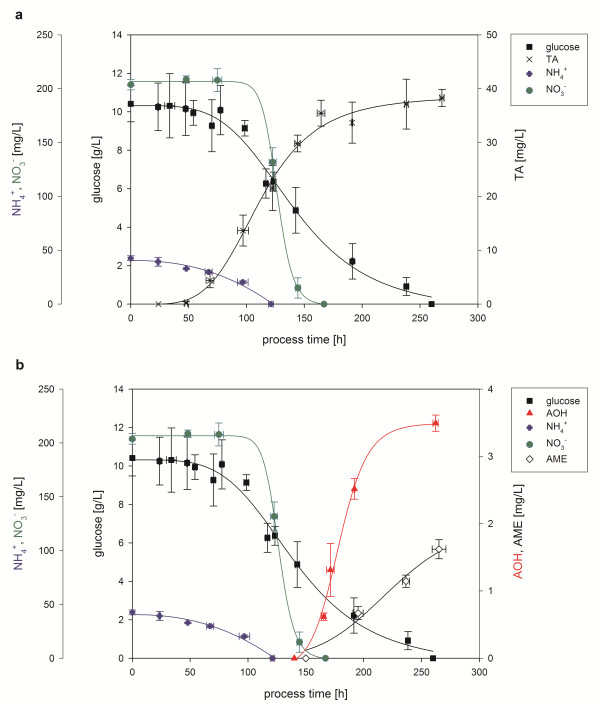
**Production of the mycotoxins tenuazonic acid (TA) (**a**), alternariol (AOH) and alternariol monomethylether (AME) (**b**) and consumption of the nutrients glucose, nitrate and ammonium (**a**, **b**) with *A. alternata *DSM 12633 in a 2 L bioreactor**. Measured glucose, nitrate, ammonium, TA, AOH and AME concentrations are given as averages of five independent fermentations. All lines represent logistic fittings of the concentrations based on Eq. 1.

To elucidate the reproducibility of the system five independent fermentations were performed. The following results represent the average data of all five fermentations (Figure 1). Consumption of the nutrients glucose, ammonium and nitrate showed characteristic logistic decrease and were fitted according to Eq. 1. The root squares for the consumption curve fittings were ≥ 0.98. Formation of the mycotoxins could be described logistically and were also fitted according to Eq. 1. The root square for the formation curve fittings of TA and AOH were ≥ 0.99 and of AME ≥ 0.97. The maintained parameters for the fittings are displayed in Table 1.

**Table 1:** Parameters of logistic fittings based on Eq.1 of nutrient consumption and mycotoxin formation in a bioreactor cultivation with *A. alternata*

	y_0_	a	x_0_	b	R^2^	Max. consumption/production rate [mg/(L*h)]
Glucose	-0.3964	10.7147	141.2869	4.2033	0.9891	84.38
Nitrate	-1.174	207.9278	126.501	18.6103	0.9997	0.742
Ammonium	0	39.0676	89.0597	4.9735	0.9828	7.67
TA	-0.0448	38.7128	112.0248	-4.5425	0.9936	0.412
AOH	-0.1304	3.6254	278.5357	-14.4819	0.9933	0.078
AME	0	2.0951	225.0668	-6.8872	0.9775	0.017

TA: tenuazonic acid; AOH: alternariol; AME: alternariol monomethylether.

Parameters were maintained from five experiments.

Both nitrogen sources and glucose were consumed completely during the process. The consumption of glucose and ammonium did not start immediately most probably due to a germination phase of approximately 24 h and the presence of yeast extract in the medium. After 50 h of cultivation first TA concentrations of 0.92 mg/L were quantified. TA production continued until the end of fermentation (260 h) but was slowed down with decreasing glucose concentrations. A maximum TA concentration of 38.28 ± 1.61 mg/L was achieved. The nitrogen sources were depleted subsequently; after total exhaustion of ammonia consumption of nitrate started. With exhaustion of nitrate first AOH concentrations could be detected and reached a maximum concentration of approximately 3.49 ± 0.12 mg/L at the end of fermentation. AME production started delayed after AOH production and reached a maximum concentration of 1.62 ± 0.14 mg/L.

**Figure 2:**
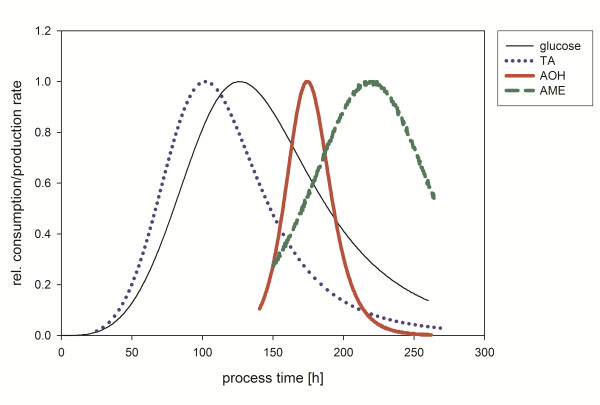
**Calculated averaged relative glucose consumption rate and mycotoxin production rates of five independent fermentations of *A. alternata *DSM 12633 in a 2 L bioreactor**. TA: tenuazonic acid; AOH: alternariol; AME: alternariol monomethylether.

Absolute consumption and production rates were obtained by derivation of the respective fitting with the maxima indicated in Table 1. All rates were normalized and the relative rates of glucose and the mycotoxins TA, AOH and AME are shown in Figure 2.

The maxima of the TA, AOH and AME production rates were calculated for 100 h, 175 h and 215 h of cultivation, respectively, whereas the maximum of the glucose consumption rate was determined for 125 h of cultivation. The maximum of glucose consumption rate indicates high metabolic activity and probably high biomass increase. Therefore, TA production appeared to be growth related while AOH and AME production seemed to be not growth-related, being this fact indicative of a typical secondary metabolite behavior.

### Influence of aeration rate on mycotoxin production

**Figure 3:**
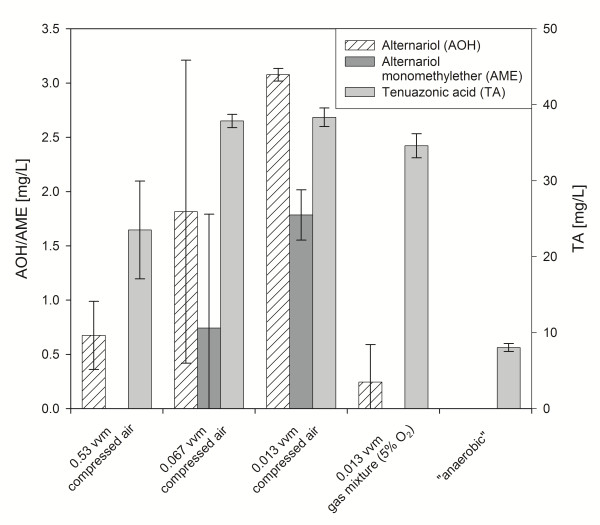
**Mycotoxin production with *A. alternata *in a 2 L bioreactor system using different aeration rates**. Results are mean of two replicates. TA: tenuazonic acid; AOH: alternariol; AME: alternariol monomethylether.

For the process development different aeration rates were tested. Figure 3 shows the effect of different aeration rates on mycotoxin production. In these experiments measurement of pO_2 _was not possible due to invasive fungal growth on the electrode. At higher aeration rates (2 vvm-0.53 vvm) *A. alternata *was not growing in pellet form, but was clinging on the flow-breaker and other fixtures very tightly. Only TA (23.52 ± 6.43 mg/L) and low concentrations of AOH (0.67 ± 0.31 mg/L) could be detected in the culture broth, AME was not detectable. A reduction of the aeration rate to 0.067 vvm resulted in an increase of all mycotoxins to 1.81 ± 1.40 mg/L AOH, 0.74 ± 1.05 mg/L AME and 37.87 ± 0.88 mg/L. A further enhancement of mycotoxin production was achieved by lowering the aeration rate to 0.013 vvm: 3.1 ± 0.06 mg/L AOH, 1.78 ± 0.23 AME and 38.35 ± 1.22 mg/L TA could be detected. Due to the decreased aeration the morphology of *A. alternata *changed: At 0.067 vvm less mycelium was clinging at the vessel wall, total biomass was reduced and pellets occurred in the culture broth. When the aeration rate was lowered to 0.013 vvm the biomass was further reduced and more mycelium was present freely in the broth in form of pellets or filaments. Inherent to the design further decrease of the aeration rate was not possible. Therefore, a gas mixture was used consisting of 5% oxygen and 95% nitrogen to decrease the oxygen supply while keeping the aeration rate at 0.013 vvm. The aeration with the gas mixture caused a drastic decrease of the polyketide mycotoxins (0.24 ± 0.35 mg/L AOH, AME was not detected) but did not affect TA production (34.60 ± 1.58 mg/L) significantly. In a final experiment the aeration was stopped completely after 48 h at 0.013 vvm (designated as "anaerobic" in Figure 3). The production of the polyketide mycotoxins seemed to be inhibited; AOH and AME were not detected, TA production was reduced to a maximum concentration of 8.04 ± 0.52 mg/L.

### Fermentation with alternative carbon and nitrogen sources

As shown previously for ochratoxin ([Bibr B1][Bibr B28]), aflatoxin (Buchanan and Stahl 1984), trichothecene (Jiao et al. 2008) and *Alternaria *toxins (Brzonkalik et al. 2011), mycotoxin production depends on nitrogen and carbon sources. In a previous study of (Brzonkalik et al. 2011) the carbon source acetate and the nitrogen source aspartic acid were promising candidates for an enhancement of *Alternaria *toxin production in static cultivation and shaking flask experiments. Consequently, fermentation experiments were performed with the described process with different combinations of carbon and nitrogen sources and are displayed in Table 2.

**Table 2:** Mycotoxin production in a 2 L bioreactor by *A.alternata *depending on carbon and nitrogen source at an aeration rate of 0.013 vvm

Carbon source	Nitrogen source	AOH [mg/L]	AME [mg/L]	TA [mg/L]	Process time [h]	BDM [g/L]
^a^Glucose	NH_4_Cl, NaNO_3_	3.49 ± 0.121	1.62 ± 0.142	38.28 ± 1.61	260	3.3 ± 0.1
^a^Glucose	Aspartic acid	7.75 ± 0.064	4.81 ± 0.014	36.54 ± 0.81	350	3.49 ± 0.27
^b^Na-acetate	NH_4_Cl, NaNO_3_	6.64 ± 0.010	ND	ND	400	1.98 ± 0.06
Na-acetate	Aspartic acid	3.66	ND	ND	400	2.62

^a ^Results are mean of 5 replicates ± standard deviation

^b ^Results are mean of 2 replicates ± standard deviation.

Acetate fermentations were conducted without pH control, glucose fermentations were performed at pH 5.5. Values display maximal detected mycotoxin concentration. Process time gives the earliest time point when the maximum concentration was achieved.

AOH: alternariol; AME: alternariol monomethylether; TA: tenuazonic acid; BDM: biodrymass; ND: below detection limit of < 0.001 mg/L.

The exchange of ammonium and nitrate for aspartic acid resulted in a 2.2 fold increase of the AOH maximum concentration to 7.75 mg/L and enhanced AME production to 4.81 mg/L. The maximum TA concentration was not affected compared to the fermentation with ammonium and nitrate. While the biomass concentration was not altered significantly, process time had to be prolonged to 350 h to reach the above mentioned mycotoxin concentrations.

The exchange of glucose for acetate in combination with ammonium and nitrate seemed to inhibit the formation of TA and AME. Only AOH was detected and its maximum concentration was enhanced 1.9 fold to 6.64 mg/L. Biomass production was decreased to 1.98 g/L, but the process was slowed down again and had to be prolonged to nearly 400 h. This may be explained by the slow consumption of acetate which took 300 h to total depletion. Keeping the pH at 5.5 in the acetate fermentation did result in an inhibition of conidia germination; therefore, the initial pH was set to 6.5 and was not controlled throughout the process. A pH optimization of this fermentation could probably reduce fermentation time. A combination of acetate and aspartic acid did not result in any further increase of the maximum AOH concentration compared to the combination of glucose and ammonium/nitrate, but again TA and AME were not detected.

When AOH content is normalized to biomass (expressed as mg mycotoxin per g biomass) maintained concentrations were the following: 1.06 mg/g (glucose/ammonium and nitrate), 2.22 mg/g (glucose/aspartic acid), 3.35 mg/g (acetate/ammonium and nitrate) and 1.40 mg/g (acetate/aspartic acid).

## Discussion

As it was shown with our results *Alternaria *toxins can be produced reproducibly in a bioreactor system under controlled conditions. Consumption of nutrient and mycotoxin formation can be characterized with logistic equations. The semi-synthetic Czapek-Dox broth is perfectly suitable for the elucidation of nutritional influences as it was shown previously by (Brzonkalik et al. 2011). Therefore, this medium was chosen for the fermentation experiments, but a further enhancement of mycotoxin production can be achieved by using other complex media.

Literature about mycotoxin production in bioreactor systems is rare; most studies were conducted in shaking flasks or solid media which cannot ensure optimal mixing, pH control and uniform supply with nutrients. Regulation of mycotoxin formation is very complex; fungal morphology and culture conditions have a great impact on mycotoxin production. As shown by (Brzonkalik et al. 2011) mycotoxin formation was different in static and in shaken culture although the same production strain and the same medium were used. Several different nitrogen and carbon sources were tested but whether mycotoxin production was higher in static or in shaken cultivation differed with each tested C or N source. With respect to the basal modified Czapek-Dox medium containing glucose and ammonium/nitrate cultivation in a bioreactor seems to be favorable since the maintained AOH concentrations are ~3 fold higher than in the shaking flask experiments mentioned by (Brzonkalik et al. 2011) and the standard deviations of detected mycotoxin concentrations were lower. However, biomass detection during the process remained difficult. The mycelium was not dispersed homologously in the culture broth. Therefore, reliable biomass determination during sampling was not possible and total biomass could only be quantified at the end of the process. Nevertheless, glucose consumption showed a typical logistic lapse which may be used as an indirect method for biomass determination as suggested for mammalian cells growing in packed-bed reactors (Meuwly et al. 2007). In case of *Alternaria *fermentations less comparable data exist. To the knowledge of the authors only one process in a stirred tank reactor was described in literature by (Hellwig et al. 2002) that reported the production of the new antibiotic altersetin. Due to structure similarities the author presumed that altersetin might be a derivative of TA. Furthermore, its formation was inhibited when nitrogen was restricted. Formation of TA during their process was mentioned but detected concentrations were not given. Optimization of stirrer speed and aeration rate in the bioreactor enhanced altersetin production considerably from 1.5 mg/L up to 25 mg/L. Bioreactor experiments do therefore not only provide more constant results, they offer also the possibility to study more parameters compared to shaking flasks, e.g. aeration. The aeration rate influences fungal morphology directly ([Bibr B27][Bibr B36][Bibr B46][Bibr B53]) and fungal morphology in turn plays an important role in metabolism during fermentation ([Bibr B10][Bibr B29]). As shown in this study, decreased aeration rates led to an increase of free mycelium in form of pellets or filaments and to higher mycotoxin concentrations. The optimal aeration rate was found to be 0.013 vvm in combination with an agitation rate of 900 rpm. A high agitation was necessary in combination with low aeration rates to prevent blocking of the air sparger due to fungal growth. For altersetin production a higher aeration rate (0.3 vvm) combined with lower agitation (100 rpm) was found to be optimal, but altersetin concentrations were not given for lower or higher agitation rates (Hellwig et al. 2002). Aflatoxin production with *Aspergillus flavus *was optimized by testing aeration rates at a constant stirring speed of 100 rpm (Hayes et al. 1966). The authors detected a nearly 20 fold increase in aflatoxin production when the aeration was enhanced from 0.6 vvm to 0.9 vvm. A further increase to 1.2 vvm resulted in decreasing aflatoxin concentrations. The effect of two aeration rates (0.5 vvm and 0.05 vvm) on fumonisin production in *Fusarium proliferatum *was studied by (Keller et al. 1997). The higher aeration with 0.5 vvm at an agitation with 500 rpm caused an increase of fumonisin production compared to the lower aeration rate. Although the comparability between all these studies is limited, it can be stated that the aeration rate has a considerable impact on fungal metabolites production. With respect to the optimal aeration rate for mycotoxin production a general statement cannot be given, but all studies found aeration rate below 1.0 vvm to be supportive for their processes, but for each process agitation has to be taken into account. An enhanced stirrer speed results in an increased dispersity of gas bubbles and therefore in an increased oxygen transfer rate and dissolved oxygen content in the culture broth. Consequently, high agitation rates enable lower aeration rates. Additionally, the type of stirrer influences shear forces and gas dispersity, but stirrer types were not specified in the above mentioned studies.

As mentioned before, mycotoxin regulation is complex and many factors are influencing their formation, including nutritional factors. Mycotoxin production is affected by carbon and nitrogen metabolism mediated by global regulators like the Cys_2_His_2 _zinc finger transcription factors AreA (nitrogen metabolism) and CreA (carbon metabolism) and their homologues. Additionally, availability of precursor units for mycotoxin production may play a role (Yu and Keller 2005). However, regulation mechanisms of *Alternaria *toxin formation are not known and the biosynthetic gene clusters have not been identified yet.

Nevertheless, acetate serves as a precursor for all three mycotoxins: TA is formed from isoleucine and acetate ([Bibr B47][Bibr B48]), the polyketides AOH and AME develop from a head to tail condensation of one molecule acetyl-CoA and six molecules of malonyl-CoA followed by a subsequent cyclization (Gatenbeck and Hermodsson 1965). Unsurprisingly, fermentation with acetate resulted in highest AOH concentration when normalized to biomass but inhibited formation of TA and AME. As shown in Figure 2 production of TA production appears to be growth-related in contrast to the polyketide mycotoxins. Simply feeding of precursor units did not enhance but inhibit TA production indicating further regulation mechanisms. The fact that acetate allows AOH but not AME production indicates for an independent regulation of the polyketide synthase and the methyltransferase enzyme which catalyzes the methylation reaction of AOH to AME (Stinson and Moreau 1986). An independent regulation of both enzymes was already presumed by (Orvehed et al. 1988). From a biotechnological point of view the possibility to produce single mycotoxins is desirable because less purification steps are necessary.

Considering the results of this study a defined process was successfully established enabling the elucidation of the effect of aeration rate, carbon and nitrogen sources on mycotoxin production. The presented process suits perfectly for further investigations of parameters influencing mycotoxin production and facilitates the comparability of different experiments.

## Competing interests

The authors declare that they have no competing interests.
